# Supplementary material for: Intra-hospital microbiome variability is driven by accessibility and clinical activities
Source: Microbiol Spectr. 2024 Jun 28;12(8):e00296-24. doi: 10.1128/spectrum.00296-24 (PMC11302010; doi:10.1128/spectrum.00296-24)
Supplement: Supplemental figures — Fig. S1 to S12. [file spectrum.00296-24-s0001.docx]

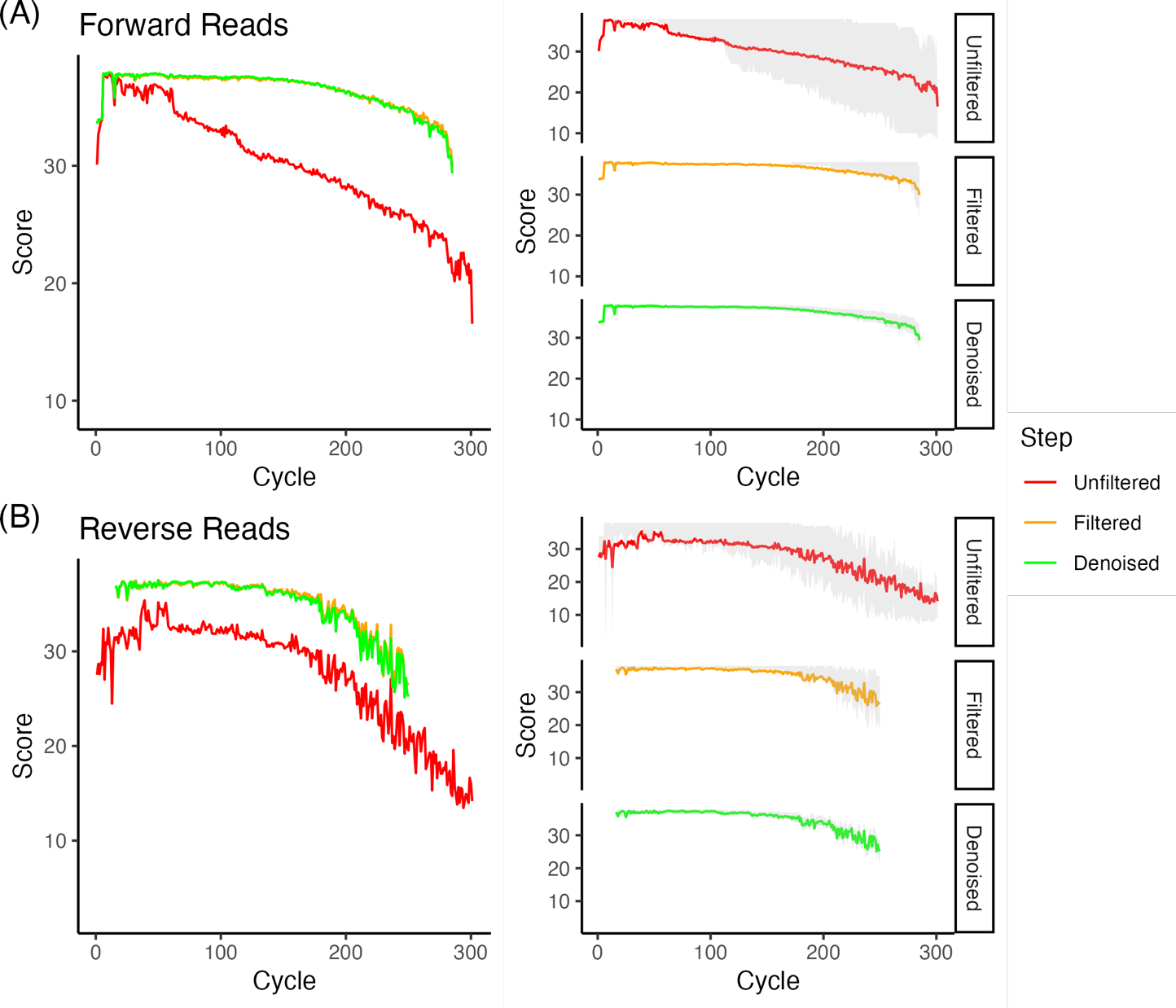


**FIG** S1 Average read quality across all reads at each base during quality steps. The shaded region represents the 25th to 75th quartile range.


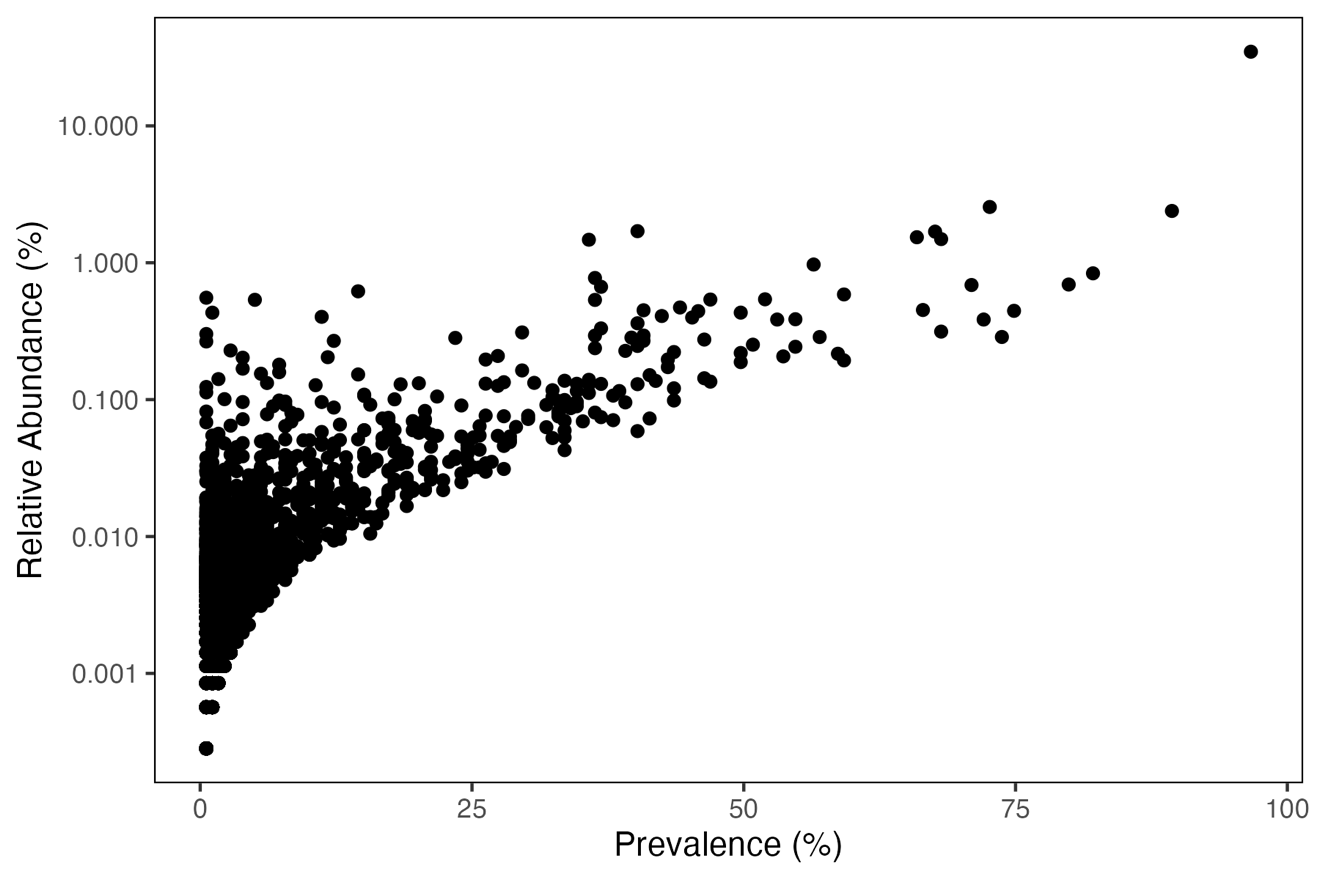


**FIG** S2 Prevalence and abundance of each ASV in all samples which exhibits a positive correlation and justifies the core-satellite model. The core population was defined as the ASVs present in at least 50% of the samples which yielded 25 ASVs which constitute 52.8% of the total relative abundance.


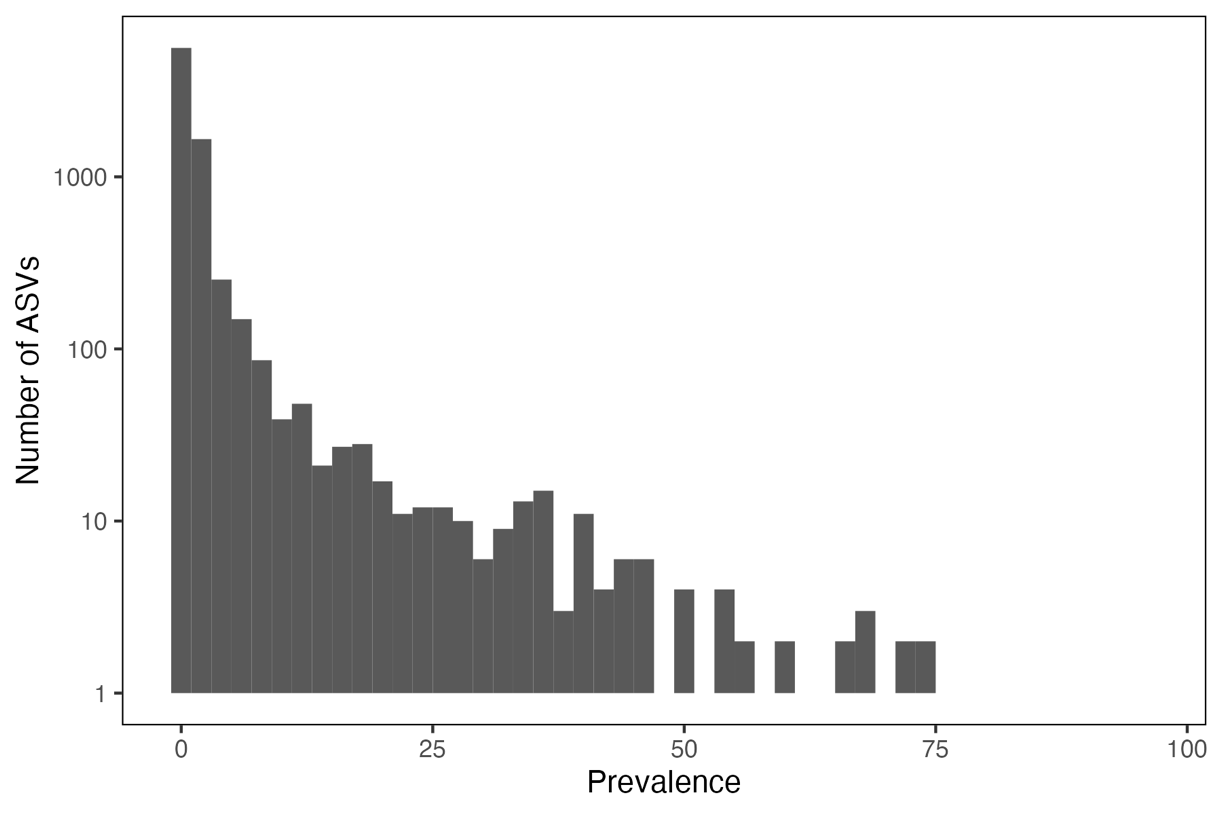


**FIG** S3 Histogram of the log-transformed prevalence of ASVs in all samples. The core population was defined as the ASVs present in at least 50% of the samples which yielded 25 ASVs which constitute 52.8% of the total relative abundance.


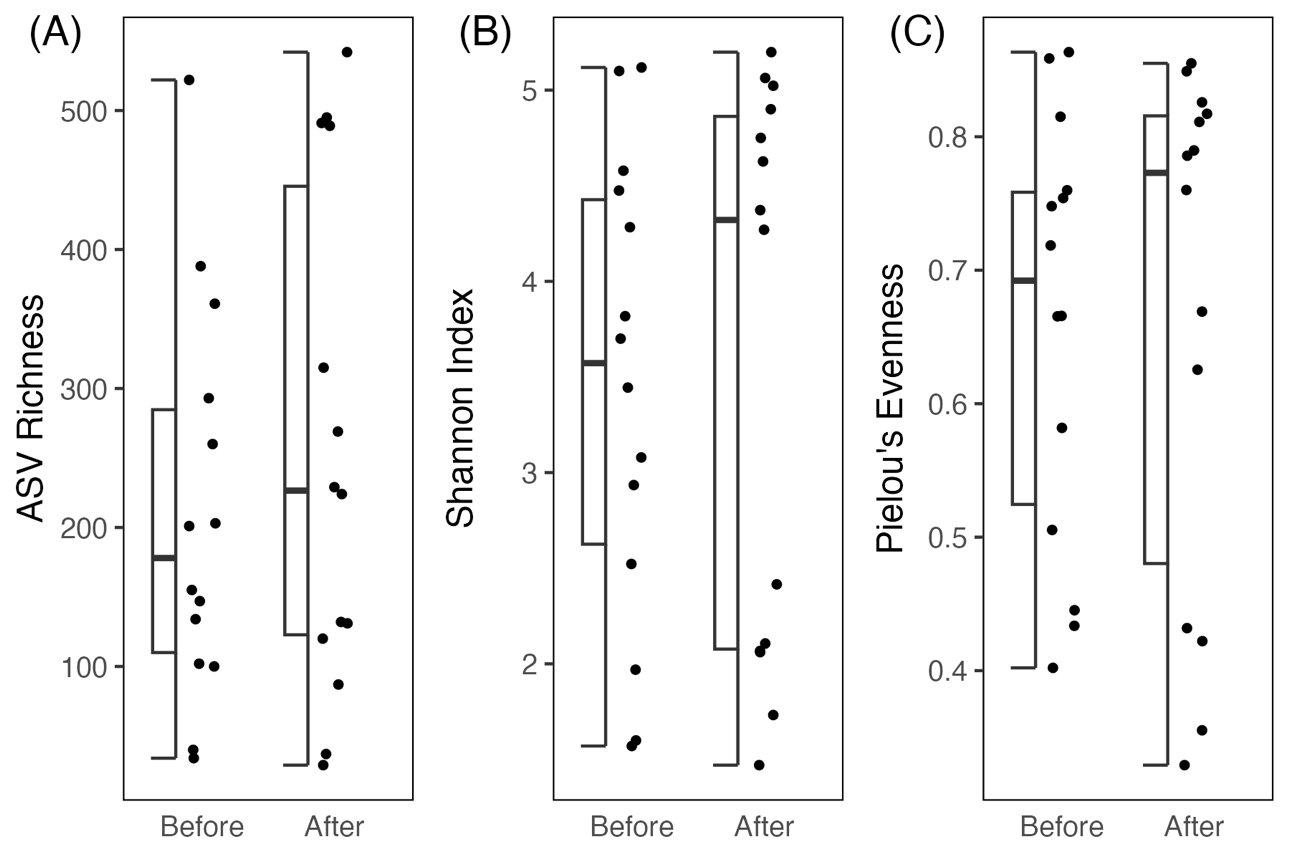


**FIG** S4 Alpha diversity in NICU patient room samples taken before (n = 14) and after (n = 14) the hospital opened for inpatient care. A) ASV richness, B) Shannon index, and C) Pielou’s evenness.


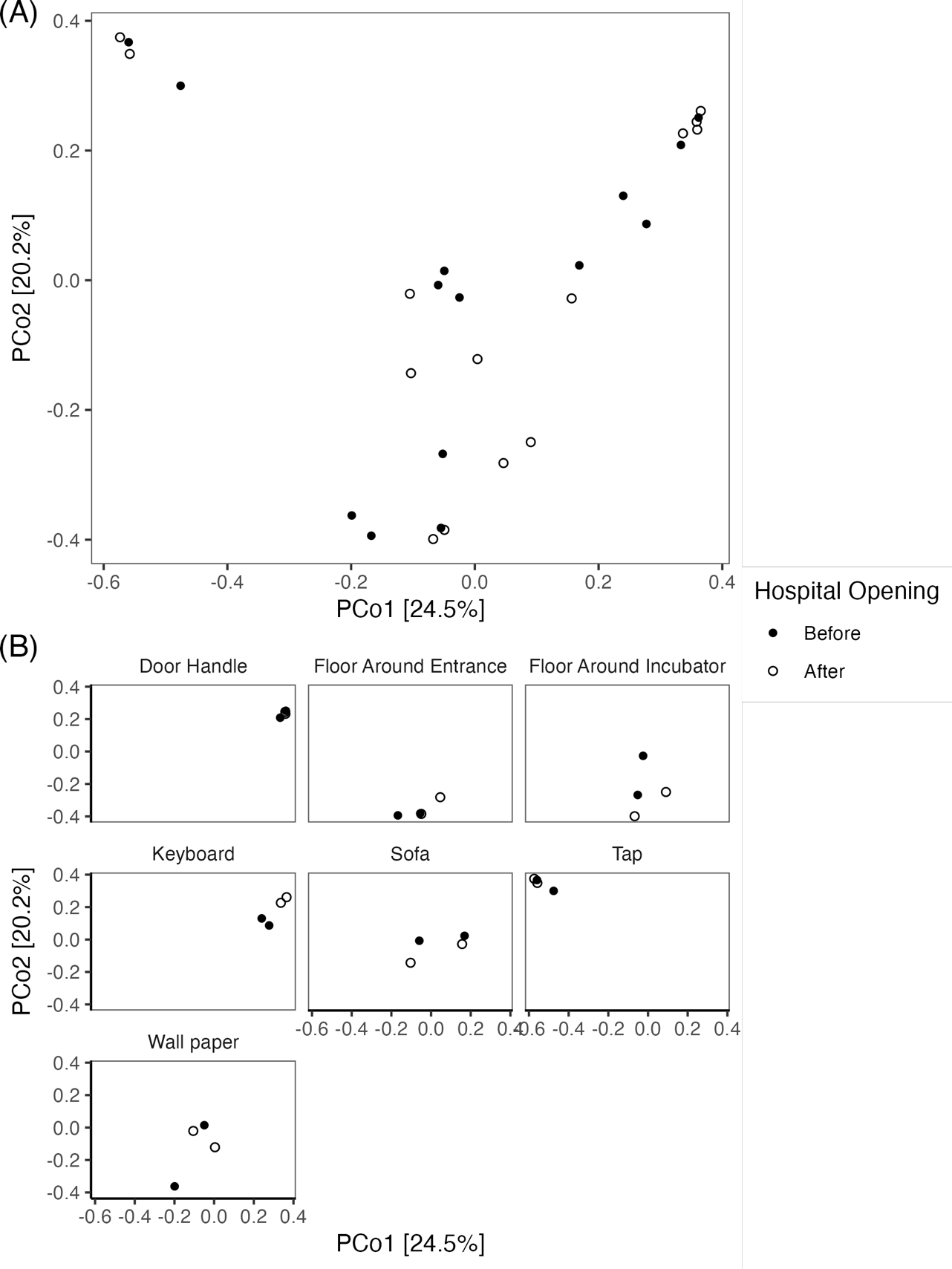


**FIG** S5 Principal coordinate analysis (PCoA) with Bray-Curtis dissimilarity of all NICU patient room samples and color coded by if the sample was taken before (n = 14) or after (n = 14) the hospital opened for inpatient care. PERMANOVA test: Ho: The centroids of the groups are the same. Ha: The centroids of the groups are not the same. p = 0.647. Ho cannot be rejected. A) includes all samples, and B) facets the plot by the surface description.


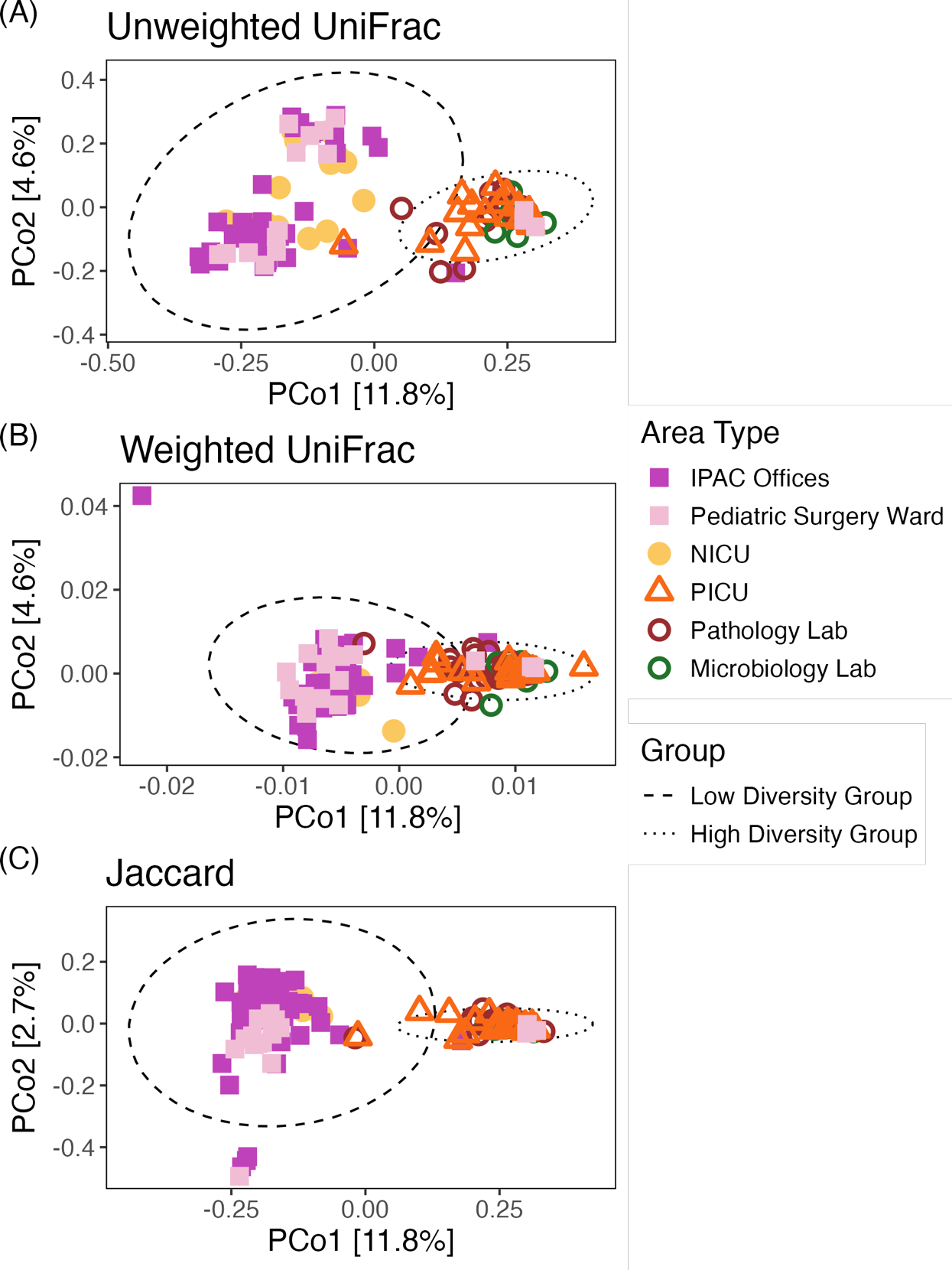


**FIG** S6 PCoA plots of all door handle, keyboard, and office electronic samples taken after the hospital opened for inpatient care (n = 120) and color coded by the area type of where the sample was taken including multivariate t distribution ellipses of the low and high diversity groups. Sample sizes: Pathology lab: n = 20, IPAC offices: n = 42, Microbiology lab: n = 8, PICU: n = 19, NICU: n = 15, Pediatric surgery ward: n = 16. PERMANOVA test: Ho: The centroids of the groups are the same. Ha: The centroids of the groups are not the same. p = 0.001. Ho can be rejected. Solid symbols are area types in the low diversity group, and open symbols are in the high diversity group. A) Unweighted Unifrac distance, B) Weighted Unifrac distance, C) Jaccard distance


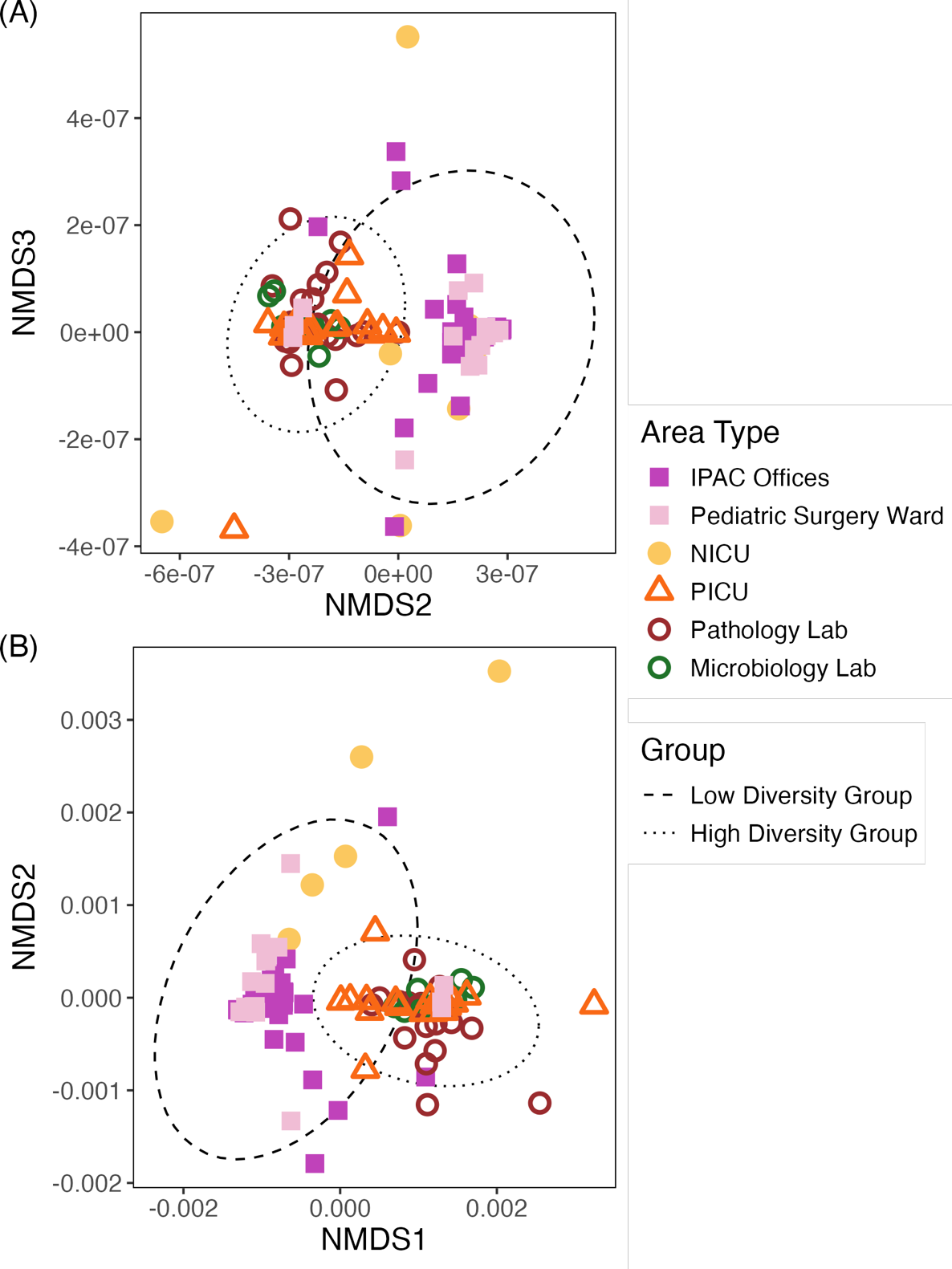


**FIG** S7 NMDS plots based on Bray-Curtis distance of all door handle, keyboard, and office electronic samples taken after the hospital opened for inpatient care (n = 120) and color coded by the area type of where the sample was taken including multivariate t distribution ellipses of the low and high diversity groups. Sample sizes: Pathology lab: n = 20, IPAC offices: n = 42, Microbiology lab: n = 8, PICU: n = 19, NICU: n = 15, Pediatric surgery ward: n = 16. PERMANOVA test: Ho: The centroids of the groups are the same. Ha: The centroids of the groups are not the same. p = 0.001. Ho can be rejected. Solid symbols are area types in the low diversity group, and open symbols are in the high diversity group. A) NMDS completed with all samples and three dimensions, showing the second and third axes, and B) NMDS with one outlier sample removed and two dimensions. ASV relative abundances were square-root transformed prior to calculating the Bray-Curtis dissimilarity.


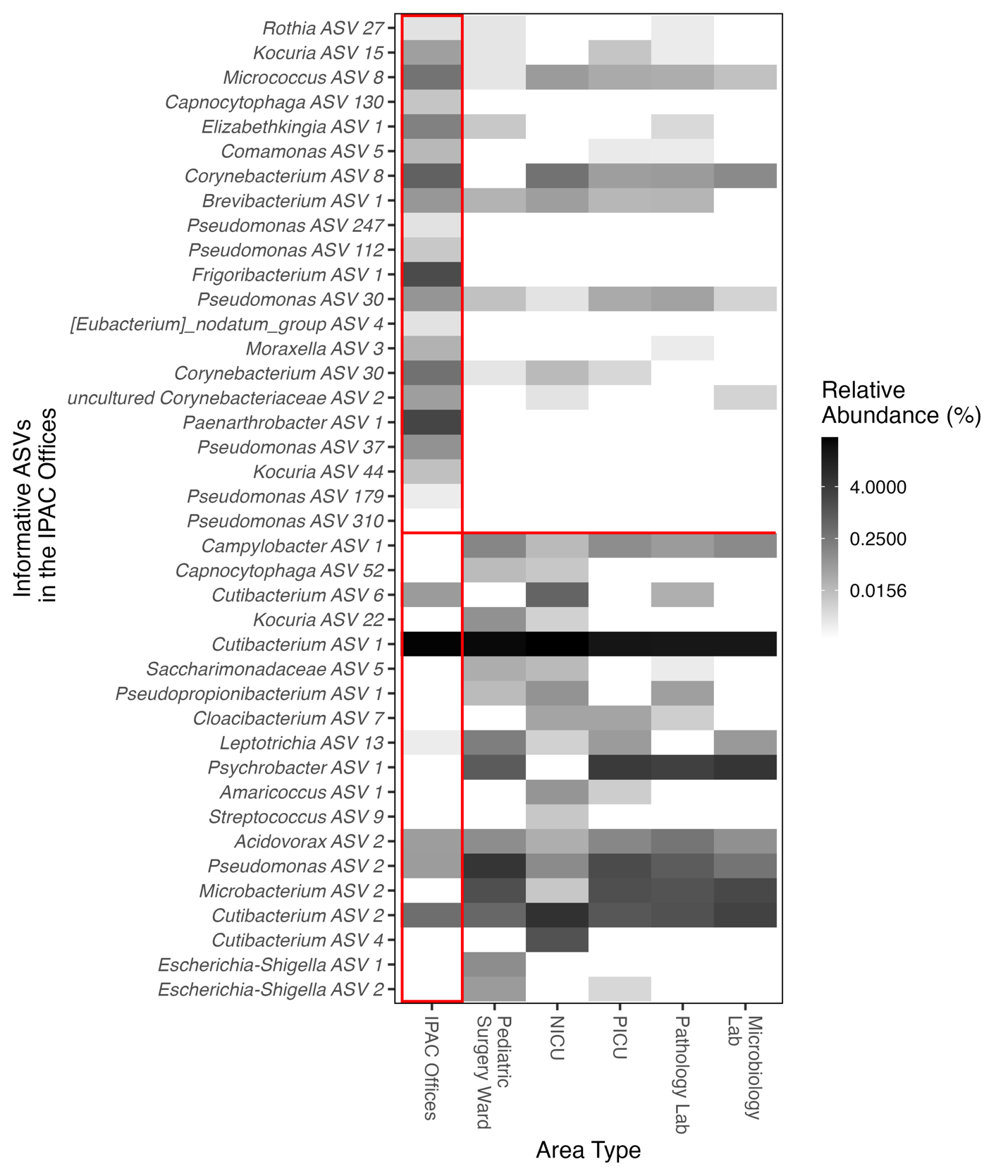


**FIG** S8 Heatmap of ASVs identified in the LASSO classification for the IPAC Offices area. Leave-one-out cross-validation was used to predict if a sample was taken from the IPAC Offices or another area based on the log-transformed relative abundance of ASVs. The ASVs are ordered from most positive to most negative coefficient. Those above the red line are associated with the IPAC Offices, and those underneath the red line are associated with other area types.


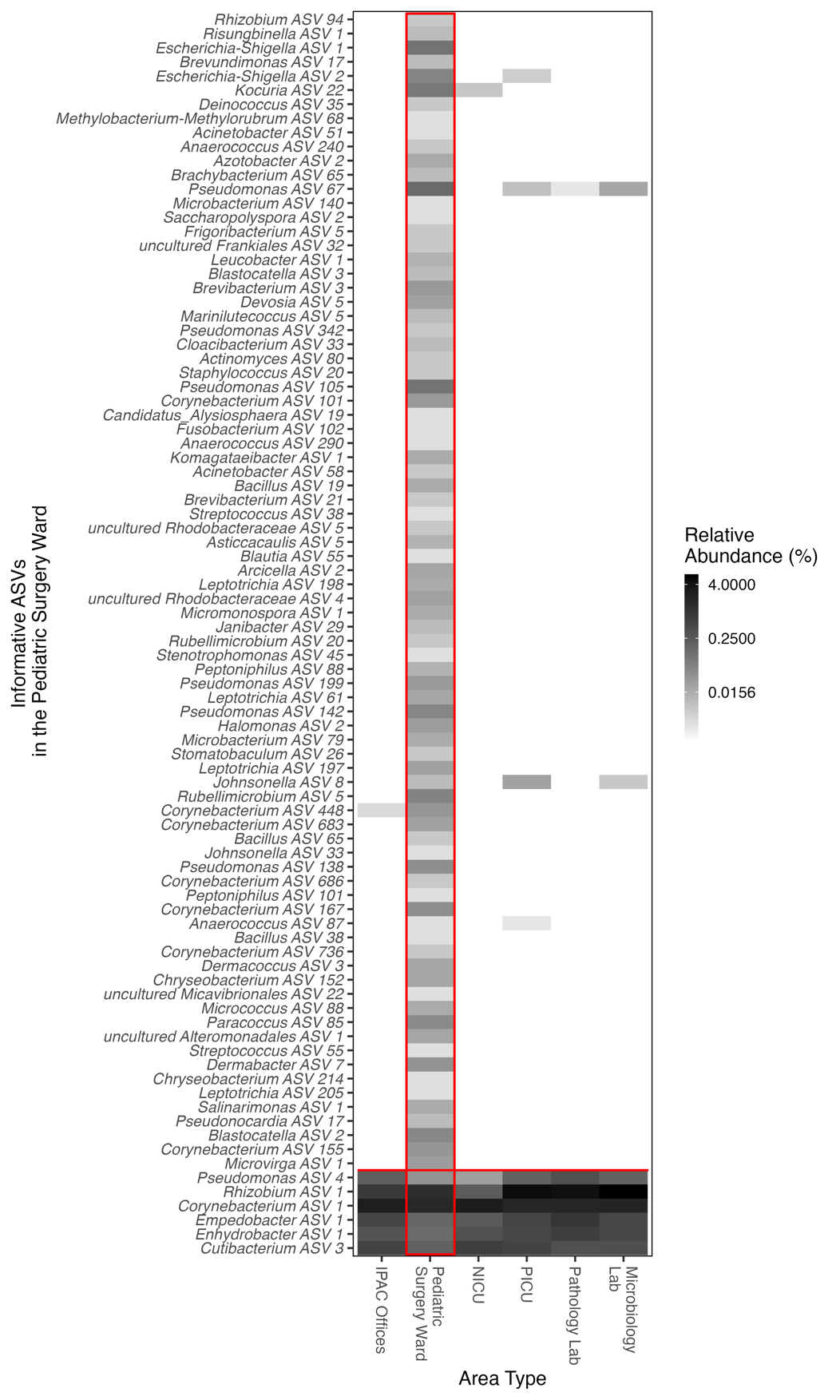


**FIG** S9 Heatmap of ASVs identified in the LASSO classification for the Pediatric Surgery Ward area. Leave-one-out cross-validation was used to predict if a sample was taken from the Pediatric Surgery Ward or another area based on the log-transformed relative abundance of ASVs. The ASVs are ordered from most positive to most negative coefficient. Those above the red line are associated with the Pediatric Surgery Ward, and those underneath the red line are associated with other area types.


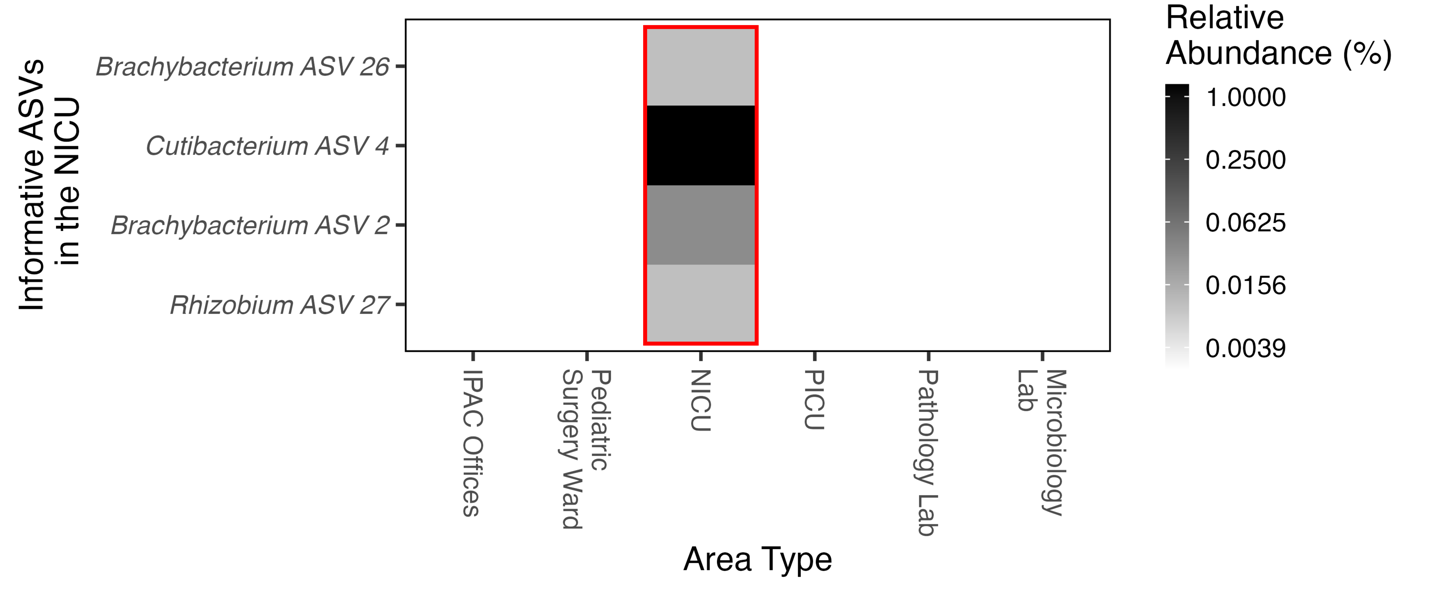


**FIG** S10 Heatmap of ASVs identified in the LASSO classification for the NICU area. Leave-one-out cross-validation was used to predict if a sample was taken from the NICU or another area based on the log-transformed relative abundance of ASVs. The ASVs are ordered from most positive to most negative coefficient.


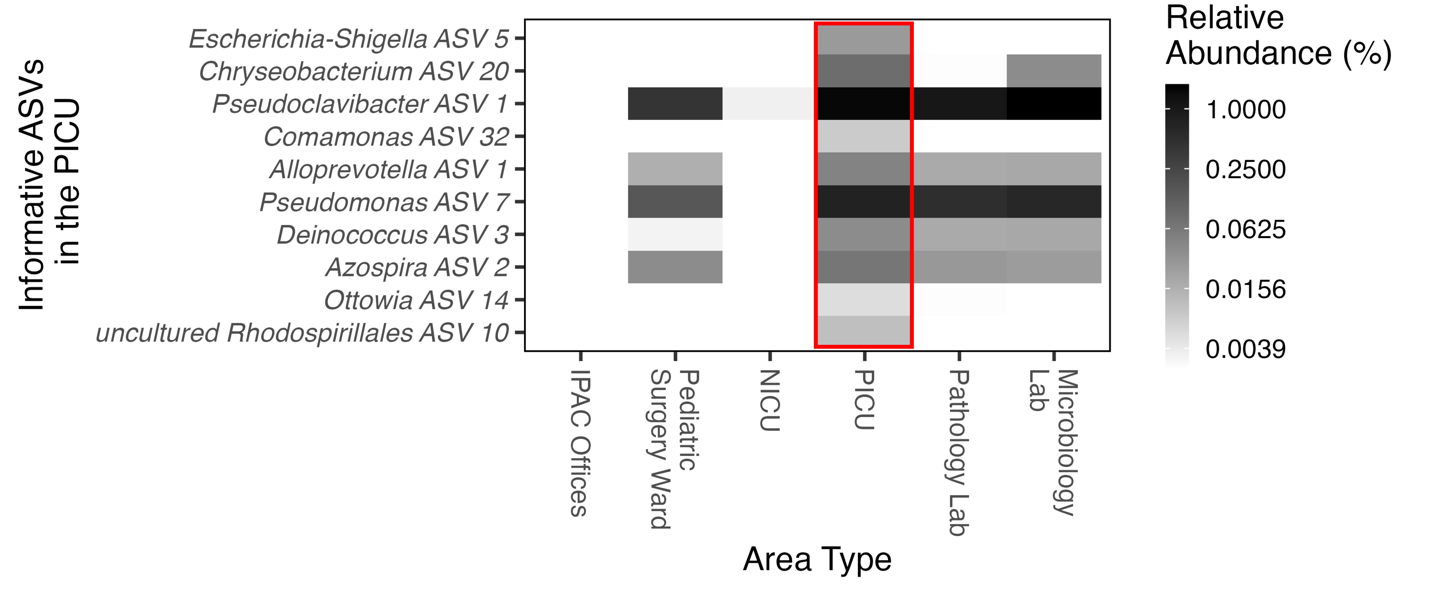


**FIG** S11 Heatmap of ASVs identified in the LASSO classification for the PICU area. Leave-one-out cross-validation was used to predict if a sample was taken from the PICU or another area based on the log-transformed relative abundance of ASVs. The ASVs are ordered from most positive to most negative coefficient.


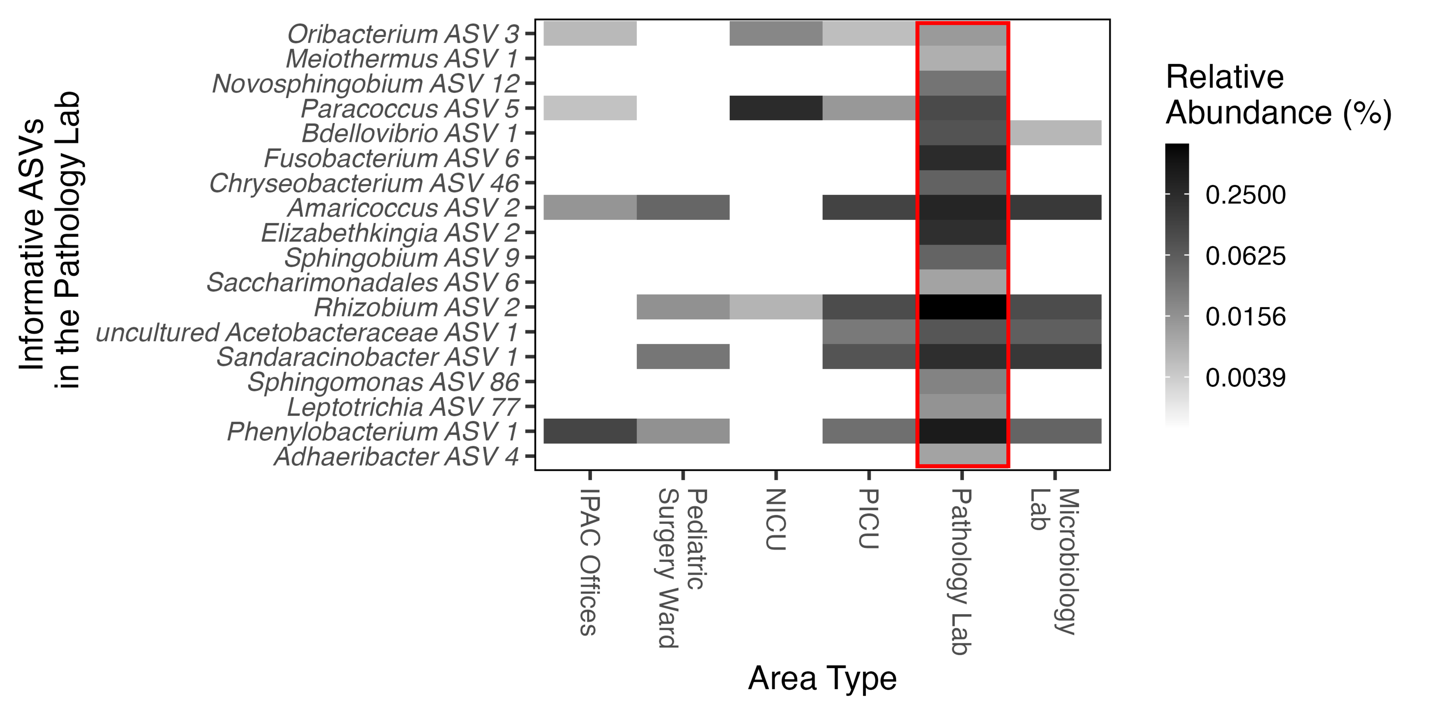


**FIG** S12 Heatmap of ASVs identified in the LASSO classification for the Pathology Lab area. Leave-one-out cross-validation was used to predict if a sample was taken from the Pathology Lab or another area based on the log-transformed relative abundance of ASVs. The ASVs are ordered from most positive to most negative coefficient.
