# Supplementary material for: Intra-hospital microbiome variability is driven by accessibility and clinical activities
Source: Microbiol Spectr. 2024 Jun 28;12(8):e00296-24. doi: 10.1128/spectrum.00296-24 (PMC11302010; doi:10.1128/spectrum.00296-24)
Supplement: Supplemental tables — Tables S1 to S10. [file spectrum.00296-24-s0002.docx]

# Supplementary Tables

**TABLE** S1 Average Denoising Statistics per Sample (n=235).

|  |  | **Percentage of Reads Remaining After Each Quality Step** | | | | | | | |  |
| --- | --- | --- | --- | --- | --- | --- | --- | --- | --- | --- |
| **Number of Unfiltered Reads** | | Filtered | | Denoised | | Merged | | Non-chimeric | |  |
| Average | SD | Average | SD | Average | SD | Average | SD | Average | SD | |
| $192,811$ | $244,915$ | $25.18\%$ | $14.27\%$ | $24.36\%$ | $13.92\%$ | $13.44\%$ | $10.35\%$ | $12.41\%$ | $8.78\%$ | |

**TABLE** S2 Average Read Quality Through the Dada2 Pipeline (n = 235).

|  |  |  | **Quality Score** | |  |
| --- | --- | --- | --- | --- | --- |
| **Pipeline Step** | **Number of Reads** | **Number of Bases** | Average | SD |  |
| Forward Reads |  |  |  |  |  |
| Unfiltered | $42,645,418$ | $300$ | $30.2$ | $4.61$ |  |
| Filtered | $13,725,430$ | $285$ | $36.5$ | $1.49$ |  |
| Denoised | $47,093$ | $285$ | $36.5$ | $1.63$ |  |
| Reverse Reads |  |  |  |  |  |
| Unfiltered | $42,645,418$ | $300$ | $27.6$ | $5.71$ |  |
| Filtered | $13,725,430$ | $235$ | $35.2$ | $2.84$ |  |
| Denoised | $30,227$ | $235$ | $35.0$ | $3.03$ |  |

**TABLE** S3 Sampling output before rarefraction.

| **Sample Type** | **Sample Counts** |
| --- | --- |
| ***Room Type*** |  |
| Patient room | 61 |
| Nursing Station | 32 |
| Microbiology Lab | 16 |
| Office room | 14 |
| Staff Room | 14 |
| Staff Lounge | 12 |
| Pathology Lab | 8 |
| Washroom - Female | 8 |
| Conference Room | 4 |
| Control room | 4 |
| General lab | 4 |
| Locker Room - Female | 4 |
| Locker Room - Male | 4 |
| Pantry | 4 |
| Washroom | 4 |
| Washroom - Male | 4 |
| ***Surface Category*** |  |
| Door Handle | 64 |
| Other | 44 |
| Keyboard | 37 |
| Office Electronics | 36 |
| Floor | 8 |
| Screen | 8 |
| ***Surface Description*** |  |
| Door Handle | 44 |
| Keyboard | 29 |
| Telephone | 8 |
| Computer keyboard | 4 |
| Computer Mouse | 4 |
| Cupboard | 4 |
| Disabled exit button | 4 |
| Door Handle Near Serology | 4 |
| Door Plate | 4 |
| Floor Around Entrance | 4 |
| Floor Around Incubator | 4 |
| Fridge Door Handle | 4 |
| Incubator Door Handle | 4 |
| Main door handle | 4 |
| Microscope | 4 |
| Nourishment fridge door | 4 |
| Omnicell Keyboard | 4 |
| Philips AED Screen | 4 |
| Photocopy Machine | 4 |
| Printer | 4 |
| Reception bench | 4 |
| Scanner | 4 |
| Screen | 4 |
| Signature scanner | 4 |
| Sofa | 4 |
| Tap | 4 |
| Telephone handle | 4 |
| Telephone Receiver Bench | 4 |
| Touch Top Waste Bin | 4 |
| Wall paper | 4 |
| Wrist band Printer | 4 |
| Xerox Machine Keypad | 4 |

**TABLE** S4 Pearson correlation between observed richness and time after opening.

| **Area Type** | **ρ** | **95% Confidence Interval** | **p-value** |
| --- | --- | --- | --- |
| IPAC Offices | $-0.2527$ | [-0.5169, 0.0555] | $1.06\times{10}^{-1}$ |
| Pediatric Surgery Ward | $0.5955$ | [0.1416, 0.8425] | $1.49\times{10}^{-2}$ |
| PICU | $-0.0471$ | [-0.4908, 0.4160] | $8.48\times{10}^{-1}$ |
| Pathology Lab | $0.0981$ | [-0.3601, 0.5181] | $6.81\times{10}^{-1}$ |
| Microbiology Lab | $0.2526$ | [-0.5500, 0.8126] | $5.46\times{10}^{-1}$ |

**TABLE** S5 Abundant taxa in Sidra hospital door handle, keyboard, and office electronic surfaces taken after opening (sample size = 120).

| **Taxa** | **Relative Abundance (%)** | **Standard Deviation (%)** |
| --- | --- | --- |
| **Phylum (All 27)** |  |  |
| Actinobacteriota | $5.83\times{10}^{1}$ | $2.63\times{10}^{1}$ |
| Proteobacteria | $2.74\times{10}^{1}$ | $2.20\times{10}^{1}$ |
| *Gammaproteobacteria* | $1.56\times{10}^{1}$ | $1.81\times{10}^{1}$ |
| *Alphaproteobacteria* | $1.17\times{10}^{1}$ | $1.01\times{10}^{1}$ |
| Bacteroidota | $6.45$ | $5.95$ |
| Firmicutes | $3.49$ | $2.54$ |
| Fusobacteriota | $2.81$ | $4.55$ |
| Patescibacteria | $5.14\times{10}^{-1}$ | $1.22$ |
| Campilobacterota | $3.63\times{10}^{-1}$ | $6.84\times{10}^{-1}$ |
| WPS-2 | $1.94\times{10}^{-1}$ | $2.55\times{10}^{-1}$ |
| Deinococcota | $1.81\times{10}^{-1}$ | $2.61\times{10}^{-1}$ |
| Cyanobacteria | $7.52\times{10}^{-2}$ | $1.96\times{10}^{-1}$ |
| Planctomycetota | $3.89\times{10}^{-2}$ | $8.69\times{10}^{-2}$ |
| Acidobacteriota | $3.42\times{10}^{-2}$ | $1.76\times{10}^{-1}$ |
| Bdellovibrionota | $3.34\times{10}^{-2}$ | $2.22\times{10}^{-1}$ |
| Chloroflexi | $3.34\times{10}^{-2}$ | $9.40\times{10}^{-2}$ |
| Verrucomicrobiota | $2.41\times{10}^{-2}$ | $6.70\times{10}^{-2}$ |
| Synergistota | $1.48\times{10}^{-2}$ | $6.45\times{10}^{-2}$ |
| Gemmatimonadota | $6.76\times{10}^{-3}$ | $6.09\times{10}^{-2}$ |
| Armatimonadota | $5.49\times{10}^{-3}$ | $2.35\times{10}^{-2}$ |
| Sumerlaeota | $2.96\times{10}^{-3}$ | $2.81\times{10}^{-2}$ |
| Nanoarchaeota | $2.53\times{10}^{-3}$ | $1.95\times{10}^{-2}$ |
| Dependentiae | $1.69\times{10}^{-3}$ | $1.85\times{10}^{-2}$ |
| SAR324_clade(Marine_group_B) | $1.69\times{10}^{-3}$ | $1.46\times{10}^{-2}$ |
| Myxococcota | $8.45\times{10}^{-4}$ | $9.25\times{10}^{-3}$ |
| Spirochaetota | $8.45\times{10}^{-4}$ | $6.52\times{10}^{-3}$ |
| Abditibacteriota | $4.22\times{10}^{-4}$ | $4.63\times{10}^{-3}$ |
| Elusimicrobiota | $4.22\times{10}^{-4}$ | $4.63\times{10}^{-3}$ |
| WS4 | $4.22\times{10}^{-4}$ | $4.63\times{10}^{-3}$ |
| **Family (Top 30)** |  |  |
| Propionibacteriaceae | $4.28\times{10}^{1}$ | $2.48\times{10}^{1}$ |
| Pseudomonadaceae | $9.54$ | $1.76\times{10}^{1}$ |
| Corynebacteriaceae | $7.65$ | $6.24$ |
| Weeksellaceae | $5.30$ | $5.74$ |
| Micrococcaceae | $3.81$ | $9.22$ |
| Rhizobiaceae | $3.48$ | $3.86$ |
| Moraxellaceae | $2.87$ | $2.75$ |
| Microbacteriaceae | $2.79$ | $9.12$ |
| Peptostreptococcales-Tissierellales | $2.48$ | $1.88$ |
| Comamonadaceae | $2.31$ | $3.13$ |
| Sphingomonadaceae | $2.16$ | $2.23$ |
| Rhodobacteraceae | $1.70$ | $2.41$ |
| Fusobacteriaceae | $1.69$ | $2.94$ |
| Caulobacteraceae | $1.59$ | $2.10$ |
| Acetobacteraceae | $1.53$ | $1.68$ |
| Leptotrichiaceae | $1.12$ | $2.05$ |
| Flavobacteriaceae | $8.46\times{10}^{-1}$ | $9.46\times{10}^{-1}$ |
| Beijerinckiaceae | $4.51\times{10}^{-1}$ | $6.94\times{10}^{-1}$ |
| Campylobacteraceae | $3.58\times{10}^{-1}$ | $6.85\times{10}^{-1}$ |
| Saccharimonadaceae | $3.48\times{10}^{-1}$ | $5.02\times{10}^{-1}$ |
| Lachnospiraceae | $3.22\times{10}^{-1}$ | $5.88\times{10}^{-1}$ |
| Actinomycetaceae | $2.52\times{10}^{-1}$ | $4.22\times{10}^{-1}$ |
| Oxalobacteraceae | $2.39\times{10}^{-1}$ | $5.70\times{10}^{-1}$ |
| Rhodocyclaceae | $2.20\times{10}^{-1}$ | $3.59\times{10}^{-1}$ |
| WPS-2 | $1.94\times{10}^{-1}$ | $2.55\times{10}^{-1}$ |
| Dermabacteraceae | $1.72\times{10}^{-1}$ | $4.42\times{10}^{-1}$ |
| Deinococcaceae | $1.61\times{10}^{-1}$ | $2.28\times{10}^{-1}$ |
| Brevibacteriaceae | $1.59\times{10}^{-1}$ | $4.26\times{10}^{-1}$ |
| Atopobiaceae | $1.44\times{10}^{-1}$ | $6.19\times{10}^{-1}$ |
| Nocardiaceae | $1.42\times{10}^{-1}$ | $8.73\times{10}^{-1}$ |
| **Genus (Top 30)** |  |  |
| Cutibacterium | $4.27\times{10}^{1}$ | $2.48\times{10}^{1}$ |
| Pseudomonas | $9.54$ | $1.76\times{10}^{1}$ |
| Corynebacterium | $6.45$ | $5.89$ |
| Chryseobacterium | $3.08$ | $3.53$ |
| Rhizobium | $3.03$ | $3.48$ |
| Fusobacterium | $1.69$ | $2.94$ |
| Psychrobacter | $1.66$ | $2.18$ |
| Micrococcus | $1.62$ | $1.85$ |
| Sphingobium | $1.47$ | $1.74$ |
| Microbacterium | $1.46$ | $5.11$ |
| Paracoccus | $1.45$ | $2.22$ |
| Anaerococcus | $1.31$ | $1.13$ |
| Brevundimonas | $1.27$ | $1.73$ |
| Comamonas | $1.21$ | $1.57$ |
| Cloacibacterium | $1.20$ | $3.69$ |
| Lawsonella | $1.17$ | $1.15$ |
| Roseomonas | $1.14$ | $1.29$ |
| Enhydrobacter | $1.11$ | $1.47$ |
| Leptotrichia | $1.08$ | $1.92$ |
| Rothia | $9.09\times{10}^{-1}$ | $1.14$ |
| Empedobacter | $8.57\times{10}^{-1}$ | $1.17$ |
| Paenarthrobacter | $8.28\times{10}^{-1}$ | $9.07$ |
| Acidovorax | $7.34\times{10}^{-1}$ | $2.02$ |
| Frigoribacterium | $6.59\times{10}^{-1}$ | $7.17$ |
| Capnocytophaga | $6.45\times{10}^{-1}$ | $9.23\times{10}^{-1}$ |
| Pseudoclavibacter | $6.17\times{10}^{-1}$ | $8.02\times{10}^{-1}$ |
| Peptoniphilus | $5.25\times{10}^{-1}$ | $5.17\times{10}^{-1}$ |
| Finegoldia | $4.73\times{10}^{-1}$ | $4.67\times{10}^{-1}$ |
| Kocuria | $4.22\times{10}^{-1}$ | $1.07$ |
| Sphingomonas | $3.65\times{10}^{-1}$ | $8.53\times{10}^{-1}$ |

**TABLE** S6 Pairwise Mann-Whitney U tests on means of alpha diversity metrics on the various area types in all door handle, keyboard, and office electronic samples taken after the hospital opened for inpatient care (n = 120).*1*

|  | **Observed Richness** | | **Shannon Index** | | **Pielou’s Evenness** | |
| --- | --- | --- | --- | --- | --- | --- |
| **Pairing** | p-value | p-adjusted | p-value | p-adjusted | p-value | p-adjusted |
| **Area Type** |  |  |  |  |  |  |
| Microbiology Lab vs IPAC Offices | $\boldsymbol{1.05\times}\mathbf{10}^{\mathbf{-5}}$ | $\boldsymbol{5.26\times}\mathbf{10}^{\mathbf{-5}}$ | $\boldsymbol{1.49\times}\mathbf{10}^{\mathbf{-8}}$ | $\boldsymbol{7.45\times}\mathbf{10}^{\mathbf{-8}}$ | $\boldsymbol{6.74\times}\mathbf{10}^{\mathbf{-5}}$ | $\boldsymbol{2.02\times}\mathbf{10}^{\mathbf{-4}}$ |
| Microbiology Lab vs NICU | $\boldsymbol{1.30\times}\mathbf{10}^{\mathbf{-2}}$ | $\boldsymbol{1.77\times}\mathbf{10}^{\mathbf{-2}}$ | $\boldsymbol{5.55\times}\mathbf{10}^{\mathbf{-4}}$ | $\boldsymbol{1.04\times}\mathbf{10}^{\mathbf{-3}}$ | $\boldsymbol{2.73\times}\mathbf{10}^{\mathbf{-4}}$ | $\boldsymbol{6.83\times}\mathbf{10}^{\mathbf{-4}}$ |
| Microbiology Lab vs Pathology Lab | $0.780$ | $0.835$ | $0.901$ | $0.901$ | $0.601$ | $0.693$ |
| Microbiology Lab vs Pediatric Surgery Ward | $\boldsymbol{9.44\times}\mathbf{10}^{\mathbf{-4}}$ | $\boldsymbol{1.77\times}\mathbf{10}^{\mathbf{-3}}$ | $\boldsymbol{1.66\times}\mathbf{10}^{\mathbf{-3}}$ | $\boldsymbol{2.76\times}\mathbf{10}^{\mathbf{-3}}$ | $\boldsymbol{1.59\times}\mathbf{10}^{\mathbf{-2}}$ | $\boldsymbol{2.66\times}\mathbf{10}^{\mathbf{-2}}$ |
| Microbiology Lab vs PICU | $0.652$ | $0.752$ | $0.389$ | $0.449$ | $0.938$ | $0.950$ |
| NICU vs IPAC Offices | $\boldsymbol{4.92\times}\mathbf{10}^{\mathbf{-5}}$ | $\boldsymbol{1.47\times}\mathbf{10}^{\mathbf{-4}}$ | $0.253$ | $0.316$ | $0.950$ | $0.950$ |
| NICU vs Pediatric Surgery Ward | $\boldsymbol{9.08\times}\mathbf{10}^{\mathbf{-3}}$ | $\boldsymbol{1.36\times}\mathbf{10}^{\mathbf{-2}}$ | $0.682$ | $0.731$ | $0.233$ | $0.317$ |
| Pathology Lab vs IPAC Offices | $\boldsymbol{2.66\times}\mathbf{10}^{\mathbf{-10}}$ | $\boldsymbol{3.99\times}\mathbf{10}^{\mathbf{-9}}$ | $\boldsymbol{2.11\times}\mathbf{10}^{\mathbf{-14}}$ | $\boldsymbol{3.16\times}\mathbf{10}^{\mathbf{-13}}$ | $\boldsymbol{3.39\times}\mathbf{10}^{\mathbf{-9}}$ | $\boldsymbol{5.08\times}\mathbf{10}^{\mathbf{-8}}$ |
| Pathology Lab vs NICU | $\boldsymbol{7.15\times}\mathbf{10}^{\mathbf{-4}}$ | $\boldsymbol{1.53\times}\mathbf{10}^{\mathbf{-3}}$ | $\boldsymbol{5.53\times}\mathbf{10}^{\mathbf{-6}}$ | $\boldsymbol{1.80\times}\mathbf{10}^{\mathbf{-5}}$ | $\boldsymbol{2.73\times}\mathbf{10}^{\mathbf{-6}}$ | $\boldsymbol{1.36\times}\mathbf{10}^{\mathbf{-5}}$ |
| Pathology Lab vs Pediatric Surgery Ward | $\boldsymbol{3.24\times}\mathbf{10}^{\mathbf{-5}}$ | $\boldsymbol{1.22\times}\mathbf{10}^{\mathbf{-4}}$ | $\boldsymbol{6.01\times}\mathbf{10}^{\mathbf{-6}}$ | $\boldsymbol{1.80\times}\mathbf{10}^{\mathbf{-5}}$ | $\boldsymbol{4.59\times}\mathbf{10}^{\mathbf{-4}}$ | $\boldsymbol{9.83\times}\mathbf{10}^{\mathbf{-4}}$ |
| Pathology Lab vs PICU | $0.173$ | $0.216$ | $0.120$ | $0.180$ | $0.309$ | $0.386$ |
| Pediatric Surgery Ward vs IPAC Offices | $0.965$ | $0.965$ | $0.174$ | $0.237$ | $6.28\times{10}^{-2}$ | $9.42\times{10}^{-2}$ |
| PICU vs IPAC Offices | $\boldsymbol{2.44\times}\mathbf{10}^{\mathbf{-9}}$ | $\boldsymbol{1.83\times}\mathbf{10}^{\mathbf{-8}}$ | $\boldsymbol{6.23\times}\mathbf{10}^{\mathbf{-12}}$ | $\boldsymbol{4.67\times}\mathbf{10}^{\mathbf{-11}}$ | $\boldsymbol{6.22\times}\mathbf{10}^{\mathbf{-8}}$ | $\boldsymbol{4.67\times}\mathbf{10}^{\mathbf{-7}}$ |
| PICU vs NICU | $\boldsymbol{7.96\times}\mathbf{10}^{\mathbf{-3}}$ | $\boldsymbol{1.33\times}\mathbf{10}^{\mathbf{-2}}$ | $\boldsymbol{5.19\times}\mathbf{10}^{\mathbf{-5}}$ | $\boldsymbol{1.30\times}\mathbf{10}^{\mathbf{-4}}$ | $\boldsymbol{2.31\times}\mathbf{10}^{\mathbf{-5}}$ | $\boldsymbol{8.68\times}\mathbf{10}^{\mathbf{-5}}$ |
| PICU vs Pediatric Surgery Ward | $\boldsymbol{8.69\times}\mathbf{10}^{\mathbf{-5}}$ | $\boldsymbol{2.17\times}\mathbf{10}^{\mathbf{-4}}$ | $\boldsymbol{7.43\times}\mathbf{10}^{\mathbf{-5}}$ | $\boldsymbol{1.59\times}\mathbf{10}^{\mathbf{-4}}$ | $\boldsymbol{3.49\times}\mathbf{10}^{\mathbf{-3}}$ | $\boldsymbol{6.54\times}\mathbf{10}^{\mathbf{-3}}$ |

*^1^*Bonferroni correction was used to adjust p-values, and significant p-values are bolded (α = 0.05).

**TABLE** S7 Pairwise PERMANOVA tests on centroids of PCoA on the various area types in different surface categories, area types, and days after opening in all door handle, keyboard, and office electronic samples taken after the hospital opened for inpatient care (n = 120).*1,2*

|  |  | **PERMANOVA** | | | |
| --- | --- | --- | --- | --- | --- |
| **Grouping** | Df | F value | R^2^ | p-value | p-adjusted |
| **Surface Category** |  |  |  |  |  |
| Keyboard vs Door Handle | 1 | $1.88$ | $2.14\times{10}^{-2}$ | $5.80\times{10}^{-2}$ | $8.70\times{10}^{-2}$ |
| Keyboard vs Office Electronics | 1 | $1.16$ | $1.86\times{10}^{-2}$ | $0.231$ | $0.231$ |
| Door Handle vs Office Electronics | 1 | $2.04$ | $2.29\times{10}^{-2}$ | $5.40\times{10}^{-2}$ | $8.70\times{10}^{-2}$ |
| **Area Type** |  |  |  |  |  |
| NICU vs IPAC Offices | 1 | $3.13$ | $5.38\times{10}^{-2}$ | $\boldsymbol{1.00\times}\mathbf{10}^{\mathbf{-3}}$ | $\boldsymbol{1.36\times}\mathbf{10}^{\mathbf{-3}}$ |
| NICU vs Pathology Lab | 1 | $14.7$ | $0.309$ | $\boldsymbol{1.00\times}\mathbf{10}^{\mathbf{-3}}$ | $\boldsymbol{1.36\times}\mathbf{10}^{\mathbf{-3}}$ |
| NICU vs Microbiology Lab | 1 | $16.0$ | $0.433$ | $\boldsymbol{1.00\times}\mathbf{10}^{\mathbf{-3}}$ | $\boldsymbol{1.36\times}\mathbf{10}^{\mathbf{-3}}$ |
| NICU vs PICU | 1 | $18.5$ | $0.366$ | $\boldsymbol{1.00\times}\mathbf{10}^{\mathbf{-3}}$ | $\boldsymbol{1.36\times}\mathbf{10}^{\mathbf{-3}}$ |
| NICU vs Pediatric Surgery Ward | 1 | $2.62$ | $8.28\times{10}^{-2}$ | $\boldsymbol{1.00\times}\mathbf{10}^{\mathbf{-3}}$ | $\boldsymbol{1.36\times}\mathbf{10}^{\mathbf{-3}}$ |
| IPAC Offices vs Pathology Lab | 1 | $20.4$ | $0.253$ | $\boldsymbol{1.00\times}\mathbf{10}^{\mathbf{-3}}$ | $\boldsymbol{1.36\times}\mathbf{10}^{\mathbf{-3}}$ |
| IPAC Offices vs Microbiology Lab | 1 | $16.2$ | $0.253$ | $\boldsymbol{1.00\times}\mathbf{10}^{\mathbf{-3}}$ | $\boldsymbol{1.36\times}\mathbf{10}^{\mathbf{-3}}$ |
| IPAC Offices vs PICU | 1 | $23.4$ | $0.284$ | $\boldsymbol{1.00\times}\mathbf{10}^{\mathbf{-3}}$ | $\boldsymbol{1.36\times}\mathbf{10}^{\mathbf{-3}}$ |
| IPAC Offices vs Pediatric Surgery Ward | 1 | $1.73$ | $2.99\times{10}^{-2}$ | $\boldsymbol{2.10\times}\mathbf{10}^{\mathbf{-2}}$ | $\boldsymbol{2.25\times}\mathbf{10}^{\mathbf{-2}}$ |
| Pathology Lab vs Microbiology Lab | 1 | $1.95$ | $6.99\times{10}^{-2}$ | $\boldsymbol{1.70\times}\mathbf{10}^{\mathbf{-2}}$ | $\boldsymbol{1.96\times}\mathbf{10}^{\mathbf{-2}}$ |
| Pathology Lab vs PICU | 1 | $2.11$ | $5.38\times{10}^{-2}$ | $\boldsymbol{2.00\times}\mathbf{10}^{\mathbf{-3}}$ | $\boldsymbol{2.50\times}\mathbf{10}^{\mathbf{-3}}$ |
| Pathology Lab vs Pediatric Surgery Ward | 1 | $8.62$ | $0.202$ | $\boldsymbol{1.00\times}\mathbf{10}^{\mathbf{-3}}$ | $\boldsymbol{1.36\times}\mathbf{10}^{\mathbf{-3}}$ |
| Microbiology Lab vs PICU | 1 | $1.27$ | $4.84\times{10}^{-2}$ | 0.1 | 0.1 |
| Microbiology Lab vs Pediatric Surgery Ward | 1 | $8.06$ | $0.268$ | $\boldsymbol{1.00\times}\mathbf{10}^{\mathbf{-3}}$ | $\boldsymbol{1.36\times}\mathbf{10}^{\mathbf{-3}}$ |
| PICU vs Pediatric Surgery Ward | 1 | $9.80$ | $0.229$ | $\boldsymbol{1.00\times}\mathbf{10}^{\mathbf{-3}}$ | $\boldsymbol{1.36\times}\mathbf{10}^{\mathbf{-3}}$ |

*^1^*ASV relative abundances were square-root transformed prior to calculating the dissimilarity.
*^2^*Bonferroni correction was used to adjust p-values, and significant p-values are bolded (α = 0.05).

**TABLE** S8 Hyperparameter tuned values for each machine learning model.1

| **Machine Learning Model** | Response | Tuned Hyperparameter |
| --- | --- | --- |
| LASSO | IPAC Offices | $1.87\times{10}^{-2}$ |
| LASSO | Pediatric Surgery Ward | $2.85\times{10}^{-2}$ |
| LASSO | NICU | $3.76\times{10}^{-2}$ |
| LASSO | PICU | $0.115$ |
| LASSO | Pathology Lab | $3.76\times{10}^{-2}$ |
| LASSO | Microbiology Lab | $1,000$ |
| Linear SVM | Area Type | $5.26\times{10}^{-2}$ |
| Radial SVM | Area Type | $16.0$ |
| Random Forest | Area Type | $14.2$ |

^1^Tuning was performed with 10-fold CV repeated 10 times maximizing the accuracy.

**TABLE** S9 The nonzero coefficients ASVs for each LASSO model and corresponding coefficients.1

| **ASV** | **Coefficient** |
| --- | --- |
| **IPAC Offices** |  |
| *Rothia* ASV 27 | $1.19$ |
| *Kocuria* ASV 15 | $0.783$ |
| *Micrococcus* ASV 8 | $0.728$ |
| *Capnocytophaga* ASV 130 | $0.695$ |
| *Elizabethkingia* ASV 1 | $0.638$ |
| *Comamonas* ASV 5 | $0.628$ |
| *Corynebacterium* ASV 8 | $0.616$ |
| *Brevibacterium* ASV 1 | $0.417$ |
| *Pseudomonas* ASV 247 | $0.311$ |
| *Pseudomonas* ASV 112 | $0.202$ |
| *Frigoribacterium* ASV 1 | $0.160$ |
| *Pseudomonas* ASV 30 | $0.119$ |
| *[Eubacterium]_nodatum_group* ASV 4 | $6.83\times{10}^{-2}$ |
| *Moraxella* ASV 3 | $6.37\times{10}^{-2}$ |
| *Corynebacterium* ASV 30 | $3.87\times{10}^{-2}$ |
| uncultured *Corynebacteriaceae* ASV 2 | $1.38\times{10}^{-2}$ |
| *Paenarthrobacter* ASV 1 | $3.68\times{10}^{-3}$ |
| *Pseudomonas* ASV 37 | $3.32\times{10}^{-3}$ |
| *Kocuria* ASV 44 | $2.88\times{10}^{-3}$ |
| *Pseudomonas* ASV 179 | $2.84\times{10}^{-4}$ |
| *Pseudomonas* ASV 310 | $6.99\times{10}^{-16}$ |
| *Campylobacter* ASV 1 | $-3.19\times{10}^{-4}$ |
| *Capnocytophaga* ASV 52 | $-8.05\times{10}^{-3}$ |
| *Cutibacterium* ASV 6 | $-1.47\times{10}^{-2}$ |
| *Kocuria* ASV 22 | $-5.29\times{10}^{-2}$ |
| *Cutibacterium* ASV 1 | $-6.91\times{10}^{-2}$ |
| *Saccharimonadaceae* ASV 5 | $-0.137$ |
| *Pseudopropionibacterium* ASV 1 | $-0.138$ |
| *Cloacibacterium* ASV 7 | $-0.156$ |
| *Leptotrichia* ASV 13 | $-0.159$ |
| *Psychrobacter* ASV 1 | $-0.354$ |
| *Amaricoccus* ASV 1 | $-0.360$ |
| *Streptococcus* ASV 9 | $-0.396$ |
| *Acidovorax* ASV 2 | $-0.413$ |
| *Pseudomonas* ASV 2 | $-0.454$ |
| *Microbacterium* ASV 2 | $-0.464$ |
| *Cutibacterium* ASV 2 | $-0.674$ |
| (Intercept) | $-0.734$ |
| *Cutibacterium* ASV 4 | $-0.736$ |
| *Escherichia-Shigella* ASV 1 | $-1.12$ |
| *Escherichia-Shigella* ASV 2 | $-1.83$ |
| **Pediatric Surgery Ward** |  |
| (Intercept) | $47.6$ |
| *Rhizobium* ASV 94 | $5.39$ |
| *Risungbinella* ASV 1 | $4.14$ |
| *Escherichia-Shigella* ASV 1 | $3.28$ |
| *Brevundimonas* ASV 17 | $3.14$ |
| *Escherichia-Shigella* ASV 2 | $1.71$ |
| *Kocuria* ASV 22 | $1.30$ |
| *Deinococcus* ASV 35 | $1.17$ |
| *Methylobacterium-Methylorubrum* ASV 68 | $0.727$ |
| *Acinetobacter* ASV 51 | $0.345$ |
| *Anaerococcus* ASV 240 | $0.211$ |
| *Azotobacter* ASV 2 | $0.202$ |
| *Brachybacterium* ASV 65 | $0.197$ |
| *Pseudomonas* ASV 67 | $0.126$ |
| *Microbacterium* ASV 140 | $0.118$ |
| *Saccharopolyspora* ASV 2 | $8.06\times{10}^{-2}$ |
| *Frigoribacterium* ASV 5 | $7.38\times{10}^{-2}$ |
| uncultured *Frankiales* ASV 32 | $4.45\times{10}^{-2}$ |
| *Leucobacter* ASV 1 | $3.47\times{10}^{-2}$ |
| *Blastocatella* ASV 3 | $3.02\times{10}^{-2}$ |
| *Brevibacterium* ASV 3 | $2.41\times{10}^{-2}$ |
| *Devosia* ASV 5 | $1.87\times{10}^{-2}$ |
| *Marinilutecoccus* ASV 5 | $6.72\times{10}^{-3}$ |
| *Pseudomonas* ASV 342 | $6.66\times{10}^{-3}$ |
| *Cloacibacterium* ASV 33 | $5.77\times{10}^{-3}$ |
| *Actinomyces* ASV 80 | $5.62\times{10}^{-3}$ |
| *Staphylococcus* ASV 20 | $2.04\times{10}^{-3}$ |
| *Pseudomonas* ASV 105 | $1.20\times{10}^{-3}$ |
| *Corynebacterium* ASV 101 | $1.17\times{10}^{-3}$ |
| *Candidatus_Alysiosphaera* ASV 19 | $1.00\times{10}^{-3}$ |
| *Fusobacterium* ASV 102 | $9.88\times{10}^{-4}$ |
| *Anaerococcus* ASV 290 | $5.60\times{10}^{-4}$ |
| *Komagataeibacter* ASV 1 | $4.70\times{10}^{-4}$ |
| *Acinetobacter* ASV 58 | $8.47\times{10}^{-5}$ |
| *Bacillus* ASV 19 | $6.91\times{10}^{-5}$ |
| *Brevibacterium* ASV 21 | $2.52\times{10}^{-5}$ |
| *Streptococcus* ASV 38 | $2.15\times{10}^{-5}$ |
| uncultured *Rhodobacteraceae* ASV 5 | $1.37\times{10}^{-5}$ |
| *Asticcacaulis* ASV 5 | $1.31\times{10}^{-5}$ |
| *Blautia* ASV 55 | $1.03\times{10}^{-5}$ |
| *Arcicella* ASV 2 | $6.44\times{10}^{-6}$ |
| *Leptotrichia* ASV 198 | $4.37\times{10}^{-6}$ |
| uncultured *Rhodobacteraceae* ASV 4 | $1.98\times{10}^{-6}$ |
| *Micromonospora* ASV 1 | $8.69\times{10}^{-7}$ |
| *Janibacter* ASV 29 | $3.47\times{10}^{-7}$ |
| *Rubellimicrobium* ASV 20 | $2.17\times{10}^{-7}$ |
| *Stenotrophomonas* ASV 45 | $1.34\times{10}^{-7}$ |
| *Peptoniphilus* ASV 88 | $6.95\times{10}^{-8}$ |
| *Pseudomonas* ASV 199 | $6.38\times{10}^{-8}$ |
| *Leptotrichia* ASV 61 | $5.53\times{10}^{-8}$ |
| *Pseudomonas* ASV 142 | $4.67\times{10}^{-8}$ |
| *Halomonas* ASV 2 | $3.45\times{10}^{-8}$ |
| *Microbacterium* ASV 79 | $1.34\times{10}^{-8}$ |
| *Stomatobaculum* ASV 26 | $8.76\times{10}^{-9}$ |
| *Leptotrichia* ASV 197 | $5.73\times{10}^{-9}$ |
| *Johnsonella* ASV 8 | $3.86\times{10}^{-9}$ |
| *Rubellimicrobium* ASV 5 | $1.18\times{10}^{-9}$ |
| *Corynebacterium* ASV 448 | $6.17\times{10}^{-10}$ |
| *Corynebacterium* ASV 683 | $3.58\times{10}^{-10}$ |
| *Bacillus* ASV 65 | $1.05\times{10}^{-10}$ |
| *Johnsonella* ASV 33 | $7.29\times{10}^{-11}$ |
| *Pseudomonas* ASV 138 | $1.74\times{10}^{-11}$ |
| *Corynebacterium* ASV 686 | $1.56\times{10}^{-11}$ |
| *Peptoniphilus* ASV 101 | $1.34\times{10}^{-12}$ |
| *Corynebacterium* ASV 167 | $2.94\times{10}^{-13}$ |
| *Anaerococcus* ASV 87 | $1.15\times{10}^{-13}$ |
| *Bacillus* ASV 38 | $1.01\times{10}^{-13}$ |
| *Corynebacterium* ASV 736 | $1.51\times{10}^{-14}$ |
| *Dermacoccus* ASV 3 | $8.09\times{10}^{-15}$ |
| *Chryseobacterium* ASV 152 | $7.85\times{10}^{-15}$ |
| uncultured *Micavibrionales* ASV 22 | $2.62\times{10}^{-15}$ |
| *Micrococcus* ASV 88 | $1.07\times{10}^{-15}$ |
| *Paracoccus* ASV 85 | $9.29\times{10}^{-16}$ |
| uncultured *Alteromonadales* ASV 1 | $8.53\times{10}^{-16}$ |
| *Streptococcus* ASV 55 | $4.87\times{10}^{-16}$ |
| *Dermabacter* ASV 7 | $4.51\times{10}^{-16}$ |
| *Chryseobacterium* ASV 214 | $4.20\times{10}^{-16}$ |
| *Leptotrichia* ASV 205 | $4.20\times{10}^{-16}$ |
| *Salinarimonas* ASV 1 | $3.04\times{10}^{-16}$ |
| *Pseudonocardia* ASV 17 | $2.29\times{10}^{-16}$ |
| *Blastocatella* ASV 2 | $2.16\times{10}^{-16}$ |
| *Corynebacterium* ASV 155 | $1.33\times{10}^{-16}$ |
| *Microvirga* ASV 1 | $1.31\times{10}^{-16}$ |
| *Pseudomonas* ASV 4 | $-3.38\times{10}^{-3}$ |
| *Rhizobium* ASV 1 | $-1.88\times{10}^{-2}$ |
| *Corynebacterium* ASV 1 | $-4.91\times{10}^{-2}$ |
| *Empedobacter* ASV 1 | $-7.05\times{10}^{-2}$ |
| *Enhydrobacter* ASV 1 | $-0.146$ |
| *Cutibacterium* ASV 3 | $-0.413$ |
| **NICU** |  |
| (Intercept) | $11.9$ |
| *Brachybacterium* ASV 26 | $2.89$ |
| *Cutibacterium* ASV 4 | $2.19$ |
| *Brachybacterium* ASV 2 | $1.79$ |
| *Rhizobium* ASV 27 | $1.97\times{10}^{-2}$ |
| **PICU** |  |
| (Intercept) | $6.24$ |
| *Escherichia-Shigella* ASV 5 | $2.19$ |
| *Chryseobacterium* ASV 20 | $0.567$ |
| *Pseudoclavibacter* ASV 1 | $0.298$ |
| *Comamonas* ASV 32 | $0.291$ |
| *Alloprevotella* ASV 1 | $0.114$ |
| *Pseudomonas* ASV 7 | $8.16\times{10}^{-2}$ |
| *Deinococcus* ASV 3 | $7.69\times{10}^{-2}$ |
| *Azospira* ASV 2 | $5.48\times{10}^{-2}$ |
| *Ottowia* ASV 14 | $4.62\times{10}^{-2}$ |
| uncultured *Rhodospirillales* ASV 10 | $2.37\times{10}^{-3}$ |
| **Pathology Lab** |  |
| (Intercept) | $29.9$ |
| *Oribacterium* ASV 3 | $2.44$ |
| *Meiothermus* ASV 1 | $2.38$ |
| *Novosphingobium* ASV 12 | $1.50$ |
| *Paracoccus* ASV 5 | $1.22$ |
| *Bdellovibrio* ASV 1 | $1.18$ |
| *Fusobacterium* ASV 6 | $1.07$ |
| *Chryseobacterium* ASV 46 | $0.800$ |
| *Amaricoccus* ASV 2 | $0.773$ |
| *Elizabethkingia* ASV 2 | $0.700$ |
| *Sphingobium* ASV 9 | $0.536$ |
| *Saccharimonadales* ASV 6 | $0.463$ |
| *Rhizobium* ASV 2 | $0.429$ |
| uncultured *Acetobacteraceae* ASV 1 | $0.365$ |
| *Sandaracinobacter* ASV 1 | $0.294$ |
| *Sphingomonas* ASV 86 | $0.257$ |
| *Leptotrichia* ASV 77 | $0.159$ |
| *Phenylobacterium* ASV 1 | $4.48\times{10}^{-2}$ |
| *Adhaeribacter* ASV 4 | $8.32\times{10}^{-3}$ |
| **Microbiology Lab** |  |
| (Intercept) | $-2.56$ |

^1^Positive values indicate the area type of interest, and negative values indicate other area types.

**TABLE** S10 Taxonomic Classification Comparison of Key ASVs.

|  | **Family** | | **Genus** | | **Species** | |
| --- | --- | --- | --- | --- | --- | --- |
| **ASV** | SILVA | Greengenes2 | SILVA | Greengenes2 | SILVA | Greengenes2 |
| LASSO Influential ASVs | | | | | | |
| Deinococcus ASV 35 | Deinococcaceae | Deinococcaceae | Deinococcus | Deinococcus_B | bacterium_1227R | Deinococcus_B terrestris |
| Meiothermus ASV 1 | Thermaceae | Thermaceae_405955 | Meiothermus | Meiothermus_B_405753 | uncultured Meiothermus | - |
| Risungbinella ASV 1 | Thermoactinomycetaceae | Thermoactinomycetaceae | Risungbinella | Risungbinella | Risungbinella massiliensis | Risungbinella massiliensis |
| Bdellovibrio ASV 1 | Bdellovibrionaceae | Bdellovibrionaceae | Bdellovibrio | Bdellovibrio | Bdellovibrio bacteriovorus | Bdellovibrio bacteriovorus_E |
| Rhizobium ASV 94 | Rhizobiaceae | Rhizobiaceae_A_501059 | Rhizobium | Mycoplana_499574 | - | - |
| Brevundimonas ASV 17 | Caulobacteraceae | Caulobacteraceae | Brevundimonas | Brevundimonas | - | Brevundimonas nasdae_A_487984 |
| Novosphingobium ASV 12 | Sphingomonadaceae | Sphingomonadaceae | Novosphingobium | Novosphingobium_485351 | - | - |
| Paracoccus ASV 5 | Rhodobacteraceae | Rhodobacteraceae | Paracoccus | Paracoccus | - | - |
| Oribacterium ASV 3 | Lachnospiraceae | Lachnospiraceae | Oribacterium | Oribacterium | - | Oribacterium asaccharolyticum |
| Escherichia-Shigella ASV 1 | Enterobacteriaceae | Enterobacteriaceae_A | Escherichia-Shigella | - | - | - |
| Escherichia-Shigella ASV 5 | Enterobacteriaceae | Enterobacteriaceae_A | Escherichia-Shigella | - | - | - |
| Escherichia-Shigella ASV 2 | Enterobacteriaceae | Enterobacteriaceae_A | Escherichia-Shigella | - | - | - |
| Brachybacterium ASV 2 | Dermabacteraceae | Dermabacteraceae | Brachybacterium | Brachybacterium | - | Brachybacterium muris |
| Brachybacterium ASV 26 | Dermabacteraceae | Dermabacteraceae | Brachybacterium | Brachybacterium | uncultured bacterium | Brachybacterium muris |
| Kocuria ASV 22 | Micrococcaceae | Micrococcaceae | Kocuria | Kocuria | - | Kocuria soli |
| Rothia ASV 27 | Micrococcaceae | Micrococcaceae | Rothia | Rothia | uncultured actinobacterium | Rothia endophytica |
| Fusobacterium ASV 6 | Fusobacteriaceae | Fusobacteriaceae_993521 | Fusobacterium | Fusobacterium_C | Fusobacterium_hwasookii | - |
| Cutibacterium ASV 4 | Propionibacteriaceae | Propionibacteriaceae | Cutibacterium | Cutibacterium | - | Cutibacterium acnes |
| LASSO Abundant ASVs |  |  |  |  |  |  |
| Deinococcus ASV 3 | Deinococcaceae | Deinococcaceae | Deinococcus | Deinococcus_B | Deinococcus wulumuqiensis | Deinococcus_B wulumuqiensis |
| Blastocatella ASV 2 | Blastocatellaceae | Blastocatellaceae_430966 | Blastocatella | - | - | - |
| Alloprevotella ASV 1 | Prevotellaceae | Bacteroidaceae | Alloprevotella | Alloprevotella | uncultured Bacteroidetes | Alloprevotella sp900095835 |
| Bdellovibrio ASV 1 | Bdellovibrionaceae | Bdellovibrionaceae | Bdellovibrio | Bdellovibrio | Bdellovibrio bacteriovorus | Bdellovibrio bacteriovorus_E |
| Devosia ASV 5 | Devosiaceae | Devosiaceae | Devosia | Devosia_A_502124 | - | - |
| Rhizobium ASV 2 | Rhizobiaceae | - | Rhizobium | - | - | - |
| Microvirga ASV 1 | Beijerinckiaceae | Beijerinckiaceae | Microvirga | Microvirga | - | - |
| Phenylobacterium ASV 1 | Caulobacteraceae | Caulobacteraceae | Phenylobacterium | Phenylobacterium | uncultured Alphaproteobacteria | Phenylobacterium haematophilum |
| Sandaracinobacter ASV 1 | Sphingomonadaceae | Sphingomonadaceae | Sandaracinobacter | Sandaracinobacter | Sandaracinobacter sp. | Sandaracinobacter neustonicus |
| Sphingomonas ASV 86 | Sphingomonadaceae | Sphingomonadaceae | Sphingomonas | Sphingomonas_L_486704 | - | - |
| Sphingobium ASV 9 | Sphingomonadaceae | Sphingomonadaceae | Sphingobium | Sphingobium_A_485959 | - | - |
| Novosphingobium ASV 12 | Sphingomonadaceae | Sphingomonadaceae | Novosphingobium | Novosphingobium_485351 | - | - |
| Amaricoccus ASV 2 | Rhodobacteraceae | Rhodobacteraceae | Amaricoccus | Amaricoccus | - | - |
| Paracoccus ASV 5 | Rhodobacteraceae | Rhodobacteraceae | Paracoccus | Paracoccus | - | - |
| Paracoccus ASV 85 | Rhodobacteraceae | Rhodobacteraceae | Paracoccus | Paracoccus | uncultured bacterium | Paracoccus luteus |
| Rubellimicrobium ASV 5 | Rhodobacteraceae | Rhodobacteraceae | Rubellimicrobium | Rubellimicrobium | uncultured bacterium | Rubellimicrobium mesophilum |
| uncultured Rhodobacteraceae ASV 4 | Rhodobacteraceae | Rhodobacteraceae | uncultured | - | uncultured proteobacterium | - |
| Halomonas ASV 2 | Halomonadaceae | Halomonadaceae_641030 | Halomonas | Halomonas_C_640989 | Halomonas stevensii | Halomonas_C_640989 stevensii |
| Pseudomonas ASV 7 | Pseudomonadaceae | Pseudomonadaceae | Pseudomonas | Pseudomonas_E_647464 | - | - |
| Moraxella ASV 3 | Moraxellaceae | Moraxellaceae | Moraxella | Moraxella_C_651924 | Moraxella nonliquefaciens | Moraxella_C_651924 nonliquefaciens |
| Oribacterium ASV 3 | Lachnospiraceae | Lachnospiraceae | Oribacterium | Oribacterium | - | Oribacterium asaccharolyticum |
| Johnsonella ASV 8 | Lachnospiraceae | Lachnospiraceae | Johnsonella | Johnsonella | uncultured bacterium | - |
| Chryseobacterium ASV 46 | Weeksellaceae | Weeksellaceae | Chryseobacterium | Chryseobacterium_796647 | - | - |
| Chryseobacterium ASV 20 | Weeksellaceae | Weeksellaceae | Chryseobacterium | Planobacterium | Chryseobacterium taklimakanense | Planobacterium taklimakanense |
| Elizabethkingia ASV 2 | Weeksellaceae | Weeksellaceae | Elizabethkingia | Elizabethkingia | Elizabethkingia miricola | - |
| Elizabethkingia ASV 1 | Weeksellaceae | Weeksellaceae | Elizabethkingia | Elizabethkingia | - | - |
| Comamonas ASV 5 | Comamonadaceae | Burkholderiaceae_A_592522 | Comamonas | Comamonas_F_589250 | - | - |
| Azospira ASV 2 | Rhodocyclaceae | Rhodocyclaceae | Azospira | Azospira_A | - | Azospira_A oryzae |
| Escherichia-Shigella ASV 1 | Enterobacteriaceae | Enterobacteriaceae_A | Escherichia-Shigella | - | - | - |
| Escherichia-Shigella ASV 5 | Enterobacteriaceae | Enterobacteriaceae_A | Escherichia-Shigella | - | - | - |
| Escherichia-Shigella ASV 2 | Enterobacteriaceae | Enterobacteriaceae_A | Escherichia-Shigella | - | - | - |
| Pseudomonas ASV 199 | Pseudomonadaceae | Pseudomonadaceae | Pseudomonas | - | - | - |
| Pseudomonas ASV 138 | Pseudomonadaceae | Pseudomonadaceae | Pseudomonas | Pseudomonas_A | - | - |
| Pseudomonas ASV 30 | Pseudomonadaceae | Pseudomonadaceae | Pseudomonas | Pseudomonas_E_648040 | - | - |
| Pseudomonas ASV 37 | Pseudomonadaceae | Pseudomonadaceae | Pseudomonas | Pseudomonas_E_647464 | - | - |
| Pseudomonas ASV 105 | Pseudomonadaceae | Pseudomonadaceae | Pseudomonas | Pseudomonas_A | - | - |
| Pseudomonas ASV 142 | Pseudomonadaceae | Pseudomonadaceae | Pseudomonas | - | - | - |
| Pseudomonas ASV 67 | Pseudomonadaceae | Pseudomonadaceae | Pseudomonas | Pseudomonas_B_650451 | - | - |
| uncultured Acetobacteraceae ASV 1 | Acetobacteraceae | Acetobacteraceae | uncultured | Paracraurococcus_506950 | - | - |
| Dermabacter ASV 7 | Dermabacteraceae | Dermabacteraceae | Dermabacter | Dermabacter | - | Dermabacter vaginalis |
| Brachybacterium ASV 2 | Dermabacteraceae | Dermabacteraceae | Brachybacterium | Brachybacterium | - | Brachybacterium muris |
| Pseudoclavibacter ASV 1 | Microbacteriaceae | Microbacteriaceae | Pseudoclavibacter | Pseudoclavibacter_A_383705 | Pseudoclavibacter helvolus | - |
| Frigoribacterium ASV 1 | Microbacteriaceae | Microbacteriaceae | Frigoribacterium | Frigoribacterium | uncultured bacterium | Frigoribacterium sp001424645 |
| Micrococcus ASV 8 | Micrococcaceae | Micrococcaceae | Micrococcus | Micrococcus | - | Micrococcus luteus |
| Paenarthrobacter ASV 1 | Micrococcaceae | Micrococcaceae | Paenarthrobacter | - | - | - |
| Brevibacterium ASV 1 | Brevibacteriaceae | Brevibacteriaceae | Brevibacterium | Brevibacterium | Brevibacterium casei | Brevibacterium casei |
| Kocuria ASV 22 | Micrococcaceae | Micrococcaceae | Kocuria | Kocuria | - | Kocuria soli |
| Kocuria ASV 15 | Micrococcaceae | Micrococcaceae | Kocuria | Kocuria | Kocuria palustris | Kocuria palustris |
| Brevibacterium ASV 3 | Brevibacteriaceae | Brevibacteriaceae | Brevibacterium | Brevibacterium | - | Brevibacterium paucivorans |
| Fusobacterium ASV 6 | Fusobacteriaceae | Fusobacteriaceae_993521 | Fusobacterium | Fusobacterium_C | Fusobacterium hwasookii | - |
| Leptotrichia ASV 197 | Leptotrichiaceae | Leptotrichiaceae | Leptotrichia | Pseudoleptotrichia | uncultured bacterium | - |
| Cutibacterium ASV 4 | Propionibacteriaceae | Propionibacteriaceae | Cutibacterium | Cutibacterium | - | Cutibacterium acnes |
| Corynebacterium ASV 8 | Corynebacteriaceae | Mycobacteriaceae | Corynebacterium | Corynebacterium | Corynebacterium kroppenstedtii | Corynebacterium kroppenstedtii |
| Corynebacterium ASV 683 | Corynebacteriaceae | Mycobacteriaceae | Corynebacterium | Corynebacterium | - | Corynebacterium gottingense |
| Corynebacterium ASV 448 | Corynebacteriaceae | Mycobacteriaceae | Corynebacterium | Corynebacterium | Corynebacterium aurimucosum | - |
| Corynebacterium ASV 167 | Corynebacteriaceae | Mycobacteriaceae | Corynebacterium | Corynebacterium | - | Corynebacterium phocae |
| uncultured Corynebacteriaceae ASV 2 | Corynebacteriaceae | Mycobacteriaceae | uncultured | Corynebacterium | uncultured bacterium | - |
| Corynebacterium ASV 155 | Corynebacteriaceae | Mycobacteriaceae | Corynebacterium | Corynebacterium | Corynebacterium suicordis | - |
| Corynebacterium ASV 30 | Corynebacteriaceae | Mycobacteriaceae | Corynebacterium | Corynebacterium | - | Corynebacterium appendicis |
| Corynebacterium ASV 101 | Corynebacteriaceae | Mycobacteriaceae | Corynebacterium | Corynebacterium | Corynebacterium riegelii | Corynebacterium riegelii |
| Core ASVs |  |  |  |  |  |  |
| Rhizobium ASV 1 | Rhizobiaceae_A_500471 | Rhizobiaceae | Agrobacterium | Rhizobium | - | - |
| Brevundimonas ASV 3 | Caulobacteraceae | Caulobacteraceae | Brevundimonas | Brevundimonas | Brevundimonas nasdae_A_487984 | - |
| Paracoccus ASV 2 | Rhodobacteraceae | Rhodobacteraceae | Paracoccus | Paracoccus | Paracoccus yeei | Paracoccus yeei |
| Pseudomonas ASV 8 | Pseudomonadaceae | Pseudomonadaceae | Pseudomonas_E_647464 | Pseudomonas | - | - |
| Enhydrobacter ASV 1 | Moraxellaceae | Moraxellaceae | - | Enhydrobacter | - | - |
| Anaerococcus ASV 2 | Peptoniphilaceae | Peptostreptococcales-Tissierellales | Anaerococcus | Anaerococcus | Anaerococcus nagyae | - |
| Peptoniphilus ASV 1 | Peptoniphilaceae | Peptostreptococcales-Tissierellales | Peptoniphilus_A | Peptoniphilus | Peptoniphilus_A lacydonensis | - |
| Chryseobacterium ASV 1 | Weeksellaceae | Weeksellaceae | Chryseobacterium_796647 | Chryseobacterium | Chryseobacterium hominis | Chryseobacterium hominis |
| Chryseobacterium ASV 2 | Weeksellaceae | Weeksellaceae | Chryseobacterium_796647 | Chryseobacterium | Chryseobacterium hominis | Chryseobacterium hominis |
| Empedobacter ASV 1 | Weeksellaceae | Weeksellaceae | Empedobacter_790298 | Empedobacter | Empedobacter stercoris | - |
| Comamonas ASV 1 | Burkholderiaceae_A_592522 | Comamonadaceae | Comamonas_F_589250 | Comamonas | Comamonas_F_589250 aquatica | Comamonas aquatica |
| Pseudomonas ASV 5 | Pseudomonadaceae | Pseudomonadaceae | Pseudomonas_E_648040 | Pseudomonas | Pseudomonas_E_648040 yangonensis | - |
| Pseudomonas ASV 4 | Pseudomonadaceae | Pseudomonadaceae | Pseudomonas_E_648040 | Pseudomonas | - | - |
| Pseudomonas ASV 2 | Pseudomonadaceae | Pseudomonadaceae | - | Pseudomonas | - | - |
| Roseomonas ASV 1 | Acetobacteraceae | Acetobacteraceae | Roseomonas_A_507160 | Roseomonas | - | Roseomonas gilardii |
| Cutibacterium ASV 3 | Propionibacteriaceae | Propionibacteriaceae | Cutibacterium | Cutibacterium | Cutibacterium granulosum | Cutibacterium granulosum |
| Micrococcus ASV 1 | Micrococcaceae | Micrococcaceae | Micrococcus | Micrococcus | Micrococcus luteus | - |
| Micrococcus ASV 3 | Micrococcaceae | Micrococcaceae | Micrococcus | Micrococcus | Micrococcus luteus | - |
| Corynebacterium ASV 1 | Mycobacteriaceae | Corynebacteriaceae | Corynebacterium | Corynebacterium | Corynebacterium tuberculostearicum | Corynebacterium tuberculostearicum |
| Rothia ASV 1 | Micrococcaceae | Micrococcaceae | Rothia | Rothia | Rothia sp001808955 | Rothia mucilaginosa |
| Rothia ASV 2 | Micrococcaceae | Micrococcaceae | Rothia | Rothia | Rothia dentocariosa | - |
| Fusobacterium ASV 1 | Fusobacteriaceae_993521 | Fusobacteriaceae | Fusobacterium_C | Fusobacterium | Fusobacterium_C periodonticum_D | Fusobacterium periodonticum |
| Cutibacterium ASV 1 | Propionibacteriaceae | Propionibacteriaceae | Cutibacterium | Cutibacterium | Cutibacterium acnes | - |
| Cutibacterium ASV 2 | Propionibacteriaceae | Propionibacteriaceae | Cutibacterium | Cutibacterium | Cutibacterium modestum | Propionibacterium humerusii |
| Lawsonella ASV 1 | Mycobacteriaceae | Corynebacteriaceae | Lawsonella | Lawsonella | - | - |
